# A π-Halogen Bond of Dibenzofuranones with the Gatekeeper Phe113 in Human Protein Kinase CK2 Leads to Potent Tight Binding Inhibitors

**DOI:** 10.3390/ph11010023

**Published:** 2018-02-17

**Authors:** Alexander Schnitzler, Andreas Gratz, Andre Bollacke, Michael Weyrich, Uwe Kuckländer, Bernhard Wünsch, Claudia Götz, Karsten Niefind, Joachim Jose

**Affiliations:** 1 Institut für Biochemie, Department für Chemie, Universität zu Köln, Zülpicher Straße 47, D-50674 Köln, Germany; Alexander.Schnitzler@posteo.de (A.S.); karsten.niefind@uni-koeln.de (K.N.); 2 Institut für Pharmazeutische und Medizinische Chemie, PharmaCampus, Westfälische Wilhelms-Universität Münster, Corrensstraße 48, D-48149 Münster, Germany; gratz.andreas@gmail.com (A.G.); andre.bo@web.de (A.B.); wuensch@uni-muenster.de (B.W.); 3 Medizinische Biochemie und Molekularbiologie, Universität des Saarlandes, Kirrberger Str., Geb. 44, D-66421 Homburg, Germany; michaelweyrich@gmx.de (M.W.); claudia.goetz@uks.eu (C.G.); 4 Institut für Pharmazeutische und Medizinische Chemie, Heinrich-Heine-Universität Düsseldorf, Universitätsstraße 1, D-40225 Düsseldorf, Germany; kucklaen@uni-duesseldorf.de

**Keywords:** human protein kinase CK2, dibenzofuran, tight binding inhibitor, crystal structure, π-halogen bond, apoptosis induction

## Abstract

Human protein kinase CK2 is an emerging target for neoplastic diseases. Potent lead structures for human CK2 inhibitors are derived from dibenzofuranones. Two new derivatives, 7,9-dichloro-1,2-dihydro-8-hydroxy-4-[(4-methoxyphenylamino)-methylene]dibenzo[*b*,*d*]furan-3(2*H*)-one (**4a**) and (*E*)-1,3-dichloro-6-[(4-methoxyphenylimino)-methyl]dibenzo[*b*,*d*]furan-2,7-diol (**5**) were tested for inhibition of CK2 and induction of apoptosis in LNCaP cells. Both turned out to be tight binding inhibitors, with IC_50_ values of 7 nM (**4a**) and 5 nM (**5**) and an apparent K_i_ value of 0.4 nM for both. Compounds **4a** and **5** reduced cellular CK2 activity, indicating cell permeability. Cell viability was substantially impaired in LNCaP cells, as well as apoptosis was induced, which was not appearing in non-neoplastic ARPE-19 cells. Co-crystallization of **4a** and **5** revealed an unexpected *π*-halogen bond of the chloro substituent at C9 with the gatekeeper amino acid Phe113, leading to an inverted binding mode in comparison to parent compound **4b**, with the Cl at C6 instead, which was co-crystallized as a control. This indicates that the position of the chloro substituent on ring A of the dibenzofuran scaffold is responsible for an inversion of the binding mode that enhances potency.

## 1. Introduction

Human CK2 is an ubiquitous protein kinase, which catalyzes the phosphorylation of serine/threonine residues within a consensus sequence which is present in a remarkable number of human proteins [1]. Meanwhile, evidence for more than 430 CK2 phospho-sites have been provided and about 2300 putative phosphorylation sites have been suggested by homology to the CK2 substrate consensus sequence [2]. Despite the number of proteins which were experimentally phosphorylated by CK2 is indeed lower, the still remaining huge number of tentative substrates in combination with its constitutive cellular activity can explain pleiotropic effects of this enzyme and could be a reason for its indispensable physiological role [3]. In addition there is experimental evidence that the enzyme has multiple functions in signal transduction and metabolic pathways [4]. Studies predicting that a substantial proportion of the phosphoproteome is phosphorylated by CK2 [2], suggested that this kinase might be responsible for maintaining an essential level of cellular phosphorylation [5]. This has been supported by more recent experimental studies analyzing the CK2 related phosphoproteome [6,7,8].

The heterotetrameric CK2 consists of two catalytically active α-isoforms (either CK2α or CK2α’) and two β-subunits [9]. Upon dimerization of two β-subunits via zinc-finger domains, the α-subunits are recruited to form the quaternary complex (“holoenzyme”) with a α_2_β_2_ stoichiometry. Despite its denomination, the “regulatory” β-subunit is not directly involved in general regulating CK2 enzymatic activity, but appears to be rather responsible for substrate specificity and stabilization of the holoenzyme [10]. Up to now it is unclear how CK2 activity is regulated in vivo. Some models propose a dynamic dissociation and spatiotemporal localization as a mechanism of regulation, other reports indicate that a controlled oligomerization of CK2 molecules may be an alternative or an additional method to influence its activity [4,11,12,13,14].

CK2 hyperactivity has been shown to promote cell proliferation, angiogenesis and to suppress apoptosis—three pivotal characteristics in the development of cancer. An increased CK2 level was found in many cancer tissues [15], and in consequence it is discussed as a prognostic marker, at least in prostate cancer [16] and acute myeloid leukemia [17]. CK2 hyperactivity can be explained by an increased expression level of at least CK2α, but is not due to a mutated, and hence, hyperactive CK2 isozyme. It was shown that upregulated CK2 amplifies essential tumor-promoting signal transduction pathways, such as the Wnt-, the NF-κB- or the PI3K/Akt-pathway [3,18] by interfering “laterally” with single central, but different components of these pathways [19,20]. In addition, CK2 mediates phosphorylation of caspase recognition sequences, masks these sites and thus protects proteins from caspase cleavage. Consequently, CK2 interferes with caspase signaling and its activity inversely correlates with the rate of apoptosis [21,22]. An increased cellular level of CK2 as such is not sufficient to provoke a cancer phenotype, but it has been shown in a mouse model that cooperation of the overexpressed enzyme with other oncogenes, such as myc or tal-1 can essentially promote cancer development [23,24]. To conclude, raised CK2 activity seems to generate a cellular milieu favorable for cancer progression, consistent with a concept of “non-oncogene addiction” [25,26]. The tumor develops an undue reliance on CK2 activity to maintain the malignant phenotype [20], suggesting CK2 to be a promising molecular target for cancer therapy [27]. In xenograft models of prostate cancer, suppression of CK2 using antisense oligonucleotides indeed induced apoptosis of malignant cells, which finally resulted in complete remission of the tumor [28].

The increasing evidence that human protein kinase CK2 is supposed to be a druggable pharmacological target resulted in several drug discovery approaches. These yielded a substantial number of potent CK2 inhibitors with different chemical scaffolds [29,30,31]. The quinoline derivative silmitasertib (formerly known as CX-4945) is an orally available ATP-competitive CK2 inhibitor with high affinity (K_i_ = 0.38 nM) [32]. Silmitasertib’s antitumor effect was suggested to be caused by the selective inhibition of CK2-dependent signaling in diverse signal transduction pathways [33]. Clinical trials of phase 1 with silmitasertib were recently completed with success in patients with advanced solid tumors, breast cancer, Castleman’s disease and multiple myeloma (www.clinicaltrials.gov identifier: NCT00891280 and NCT01199718) [32,34] and it is at current in clinical trials of phase 2 in combination with cisplatin and gemcitabine for the treatment of cholangiocarcinoma (www.clinicaltrials.gov identifier: NCT02128282). The still remarkable success of silmitasertib in early drug development confirms human protein kinase CK2 to be a valid and druggable therapeutic target. This is further confirmed by another CK2 inhibitor being in clinical trials. The peptide CIGB-300 was identified to cover the phosphorylation site in the consensus sequence of CK2 substrates and thereby inhibiting CK2 activity [35]. It is at current in clinical trials phase 2 for the treatment of squamous cell carcinoma or adenocarcinoma of the cervix with local application of CIGB-300 (www.clinicaltrilas.gov identifier: NCT01639625).

**Figure 1:**
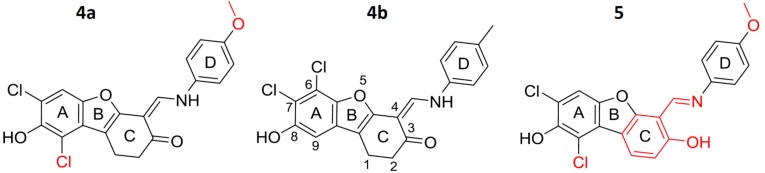
New dibenzofuran derivatives **4a** and **5** tested for CK2 inhibition. Structural differences between the compounds are given in red. Compound **4b** was published before [28,37] and was therefore used as a reference.

We have identified dibenzofurans as lead structures for potent inhibitors of human protein kinase CK2 [36]. The most potent derivative, with an IC_50_ value of 29 nM and a K_i_ value of 15 nM [37], was 6,7-dichloro-1,4-dihydro-8-hydroxy-4-[(4-methylphenylamino)methylen]dibenzo[*b*,*d*]furan-3 (2*H*)-one (**4b**, Figure 1). Compound **4b** only inhibited seven other kinases (Aurora A, KDR/VEGFR2, SGK1, FLT4/VEGFR3, PIM1, PKD2, and LCK), to similar extent as CK2, and turned out to be cell-permeable and capable of inducing apoptosis in the prostate cancer cell LNCaP. Most recently it was shown, that—depending on the salt concentration—4-carboxy-6,8-dibromo-flavonol, also a nanomolar inhibitor of CK2, forms an unusual *π*-halogen bond with an aromatic side chain of the catalytic α-subunit [38]. Because this *π*-halogen bond was found only at high concentrations of the kosmotropic salt NaCl, but not at low concentrations, and hence was dependent on a conformational change of the protein, it appeared worth investigating whether such halogen bonds could also be responsible for a positional or conformational adaption of the inhibitor to a constant protein conformation. For this purpose two derivatives of **4b** were synthesized: compounds **4a** (7,9-dichloro-1,2-dihydro-8-hydroxy-4-[(4-methoxyphenylamino)methylene]dibenzo[*b*,*d*]furan-3(2*H*)-one) and **5** ((*E*)-1,3-dichloro-6-[(4-methoxyphenylimino)methyl]dibenzo[*b*,*d*]furan-2,7-diol) (Figure 1), both containing the second chloro substituent at a different position than **4b**. In addition **4b** and **4a** contained a flexible connection between the dibenzofuran scaffold and the methoxyphenyl moiety, in contrast to **5**, in order to evaluate the impact of planarity of the four ring system on inhibition of CK2. The enzymological, cellular and structural studies with **4b**, **4a** and **5** as described herein revealed that the change of the halogen position as indicated above improves the inhibitory efficacy substantially, an effect that can be rationalized by a surprising reversal of the binding mode.

## 2. Results

### 2.1. Synthesis of **4a** and **5**

As previously reported, 1,4-benzoquinone reacted with 2-arylaminomethylenecyclohexanone derivatives (e.g., **2**, Supplementary Information Scheme S1) to afford tetrahydrocarbazoles [39]. However, chlorinated 1,4-benzoquinones provided dibenzofurans **4** under the same reaction conditions. The higher redox potential of chlorinated 1,4-benzoquinones **1a** and **1b** compared to unsubstituted 1,4-benzoquinone is responsible for the different reaction pathways, since oxidation of the enaminoketones **2a** and **2b** to provide dienaminoketones **3a** and **3b** is the first step of the transformation [39]. Reaction of dienaminoketones **3a** and **3b** with a second equivalent of 2,6- or 2,3-dichloro-1,4-benzoquinone **1a** and **1b** resulted in the dibenzofurans **4a** and **4b**, respectively [40]. The fully aromatic dibenzofuran **5** was obtained by oxidation of **4a** with 2,6-dichloro-1,4-benzoquinone (**1a**). Since the aromatic product can exist in two tautomeric forms, a 2D NMR spectrum was recorded. The NMR spectrum in the solvent DMSO clearly indicates the presence of the phenolimine tautomer **5**, because a correlation signal between the aldimine proton at 9.16 ppm and carbon atom at 154.3 ppm was detected. A signal beyond 162 ppm, which was expected for the carbonyl carbon atom of a keto enamine tautomer could not be detected. Thus, the existence of the phenolimine tautomer **5** at least in the solvent DMSO was shown unequivocally.

### 2.2. Inhibition of Human Protein Kinase CK2 In Vitro

**Figure 2:**
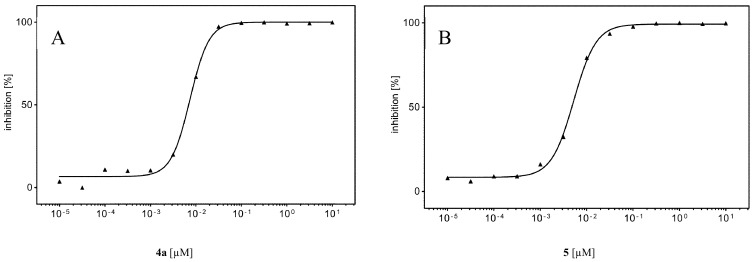
Dose-response curves for the determination of IC_50_ values of **4a** (**A**) and **5** (**B**). Human protein kinase CK2 holoenzyme was pre-incubated for 10 min with 13 different concentrations of each compounds ranging from 0.01 nM to 10 μM before the enzymatic activity was determined. The residual activity was set into relation of the activity of the enzyme without added compounds but with the same volume of DMSO, which was taken as 100%. The resulting inhibition in % was set into relation to the concentration of the corresponding compound in a logarithmic scale. For the calculation of the sigmoidal curve fitting the date GraphPad Prism (GraphPad, La Jolla, CA, USA) was used, as well as for the compound concentrations corresponding to 50% enzyme inhibition. The IC_50_ values determined this way turned out to be 7 nM for **4a** and 5 nM for **5**.

**Figure 3:**
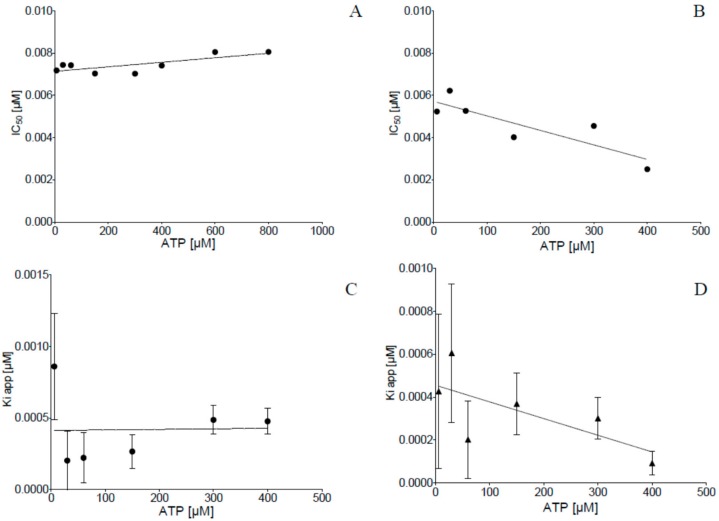
IC_50_ values of **4a** and **5** determined at different ATP-concentrations (**A**,**B**) and calculation of the apparent K_i_ values of **4a** (**C**) and **5** (**D**) by the Morrison equation using the initial reaction velocity determined for each ATP concentration used [45]. Phosphorylation of the substrate peptide RRRDDDSDDD by human protein kinase CK2 holoenzyme was determined in the CE assay at different ATP-concentrations (6, 30, 60, 150, 300, and 400 μM). As no K_i_ value was possible to determine by this strategy, the enzymatic reactions were repeated independently three times and the initial reaction velocities obtained thereby for each ATP concentration were put into the Morrison equation which enables to calculate a so-called apparent K_i_-value. For both compounds the interception of the best fit line with the Y-axis yielded a K_i app_ 0.041 nM +/− 0.02 for **4a** and 0.46 nM +/− 0.09 for **5**.

To evaluate the inhibitory effect of the two dibenzofuran derivatives **4a** and **5** on human CK2, the recombinantly expressed and purified CK2α_2_β_2_ holenzyme was used. The phosporylation of an artificial substrate peptide (RRRDDDSDDD) by the purified holoenzyme was analyzed in a capillary electrophoresis (CE) assay as described before [41]. This peptide is commonly used for the determination of CK2 activity and phosphorylation was determined in the presence and in the absence of the compounds. For this purpose a stock solution of 10 mM in DMSO was prepared for each compound and diluted in the assay to a final concentration of 10 µM. At this concentration the residual enzymatic activity was less than 1% in case of both compounds, when compared to the enzymatic activity without compounds. Consequently 13 concentrations of both dibenzofurans, ranging from 0.01 nM to 10 µM were tested on their inhibition of CK2 dependent substrate phosphorylation in the presence of 100 μM ATP. This resulted in a dose-response curve, which served for IC_50_ value determination by setting the percentage of inhibition in relation to the inhibitor concentration in a logarithmic scale (Figure 2). By this method, the IC_50_ value of **4a** was determined to be 7 nM and the IC_50_ value for **5** was determined to be 5 nM. More recently, Guerra et al. reported on a compound “D11” obtained by a screening of the Diversity Set III of the DTP program from the NCI/NIH, which appears to be identical with compound **5** in this study, and determined an IC_50_ value of 7 nM towards CK2α in a radiometric assay [42]. In our preceding investigation, the K_i_ value of **4b** (Figure 1) had been determined experimentally to be 15 nM, by applying different ATP concentrations ranging from 6 µM to 400 µM, followed by IC_50_ value determination for each of the different ATP concentrations [37]. When the same procedure was applied to **4a** and **5**, however, no linear dependency of the IC_50_ values obtained from the different ATP concentrations was detectable (Figures 3A,B), as it would have been expected for ATP-competitive inhibitors. Moreover, when the CK2 activity was determined at a constant ATP concentration of 60 µM with varying substrate concentrations at very low concentrations of **4a**, the double reciprocal plot of the data indicated a non-competitive mode of action (data not shown), which actually suggested that **4a** shows similar binding affinities for both, the free enzyme and the enzyme—substrate binary complex, in this case. As a consequence of these controversial results, we repeated the measurements with **4b** on ATP competitiveness and controlled the **4a** and **5** preparations in use for impurities, both with no other outcome than before. Because the first results coming from the co-crystallization experiments (described in detail in Section 2.6) clearly indicated an ATP-competitive binding mode of **4b** and **5**, we were confronted with the discrepancy, that such competitiveness was not possible to show in inhibition experiments with compounds **4b** and **5** under gradually increasing ATP-concentrations (Figures 3A,C). Therefore, it was taken under consideration whether **4a** and **5** could be so-called “tight-binding” inhibitors. Such a “tight-binding” effect can be observed in case the IC_50_ value of an inhibitor is in the same range as the concentration of the enzyme in the assay [43]. In our assay, the concentration of CK2 was 9 nM (concentration of active sites 18 nM, due to the α_2_β_2_ architecture) and the IC_50_ value for **4a** turned out to be 7 nM and 5 nM for **5**. In such case the population of free enzyme molecules is significantly depleted by formation of enzyme-inhibitor complex and the simplification that the concentration of the free enzyme is equal to the total enzyme concentration as required for Michaelis-Menten kinetics is no longer valid [44]. As a consequence the concentration of free enzyme varies, which has a severe influence on the IC_50_ values as determined, and the double-reciprocal plot looks like classical non-competitive inhibitor. But this is an artefact of measurement and does not reflect the “true” situation [44]. The IC_50_ value is still dependent on the ATP-concentration, but it cannot be measured in this way. Hence, for such “tight-binding” inhibitors a graphical determination of the K_i_ value using e.g., Lineweaver-Burk plot is not possible. In order to be still able to determine the inhibition constant, the Morrisson equation was developed which is a non-linear fit of the initial reaction velocities in dependence of the inhibitor concentration and which considers the contemplable concentrations of enzyme and substrate [45]. This can be used to determine the so-called apparent K_i_ (K_i app_) for different substrate (ATP) concentrations. These experiments have been performed for **4a** and **5** with ATP concentrations ranging for 6–400 µM, and the calculated K_i app_ values by the Morrission equation were plotted against the ATP concentration (Figures 3C,D) [46].

Despite the fact that for each substrate concentration the standard error of the mean (SEM) of the three independent determinations was indeed noticeable, which could have been due to the difficult measurement of the initial reaction velocity, a clear indication for a K_i app_ for both compounds was given by extrapolation of the best fit line with the Y axis (Figure 3C,D), at an ATP concentration = 0. The K_i app_ values obtained thereby were 0.41 nM +/− 0.02 for **4a** and 0.46 nM +/− 0.09 for **5**. In order to test the reliability of these values, the determination of K_i app_ at different ATP-concentrations was repeated for **4a**, but with double the amount of the enzyme CK2. This resulted in K_i app_ values at the same level of 4 nM, but the fluctuation obtained with the different ATP concentrations was much lower (data not shown). In consequence we considered **4a** and **5** to ATP competitive inhibitors of human protein kinase CK2 with a “tight binding” characteristic and with K_i app_ values in the subnanomolar range.

### 2.3. Selectivity

As **4b**, the lead compound for the development of **4a** and **5**, appeared to be relatively specific towards human protein kinase CK2, a selectivity profile study was performed by Reaction Biology Corp. (Malvern, PA, USA) for **4a** and **5** as well. For this purpose 61 kinases from different representative subgroups (and CK2α and CK2α’) were tested. Testing was performed with inhibitor concentrations of 10 µM and an ATP concentration of 10 μM. The residual enzyme activity as obtained was set in relation to a control without inhibitor. Under these inhibition conditions the residual activities obtained with **4a** and **5** was less than 2% for CK2α and less than 1% for CK2α’. As shown in Table 1, the selectivity profile obtained was surprisingly different for the three compounds, despite their pronounced structural similarities. **4a** inhibited only 6 out of the 61 kinases to more than 70% at a concentration of 10 μM. 5 of these kinases belong to different families (Aurora A, SKG1, CAMKK2, DYRKB1, and FLT3). 5 inhibited 11 kinases out of 9 different families to more than 70% at 10 µM instead (for a complete listing of inhibition data towards the 61 kinases, see supplementary Figure S2). This could reflect the lower flexibility between the rings C and D in **5**, which could overcome an entropic effect with these kinases as obtained with **4b** and **4a**. Interestingly, only two kinases, Aurora A and SGK1 were inhibited by all three dibenzofurans to more than 70%. No other kinase was inhibited to the same extent by **4a** and **4b**, indicating that selectivity of ATP-competitive inhibitors, despite depending on a quite similar active site, can be addressed with distinct modifications of an identical scaffold. This demands further investigation. Nevertheless all three compounds of the dibenzofuran type as presented here can be considered as quite selective inhibitors of human CK2.

We recently reported a whole cell CK2 activity assay, which can be used for testing the selectivity of compounds towards CK2α and CK2α’ [47]. It is based on the surface display of the human CK2 subunits, either α or α’ together with β on *E. coli*, were they are able to form a functional tetrameric holoenzymes, either ααββ or α’α’ββ, depending on which α isoform has been expressed [48]. This technique was used to investigate whether **4a** or **5** have preference to one of the two catalytic isoforms of CK2. As depicted in Supplementary Information, Figure S2, the IC_50_ values obtained for α and α’ containing holoenzymes were in the same order of magnitude for **4a** and **5**. There was a tendency to for stronger inhibition of the CK2α’ containing holoenzyme in comparison to CK2α containing holoenzyme by a factor of 1.5 for both compounds. However, this appeared to be not statistically significant. Nevertheless, a similar tendency has been observed during the selectivity testing with the kinase panel (Supplementary Information Figure S2).

### 2.4. Effects of the Dibenzofuran Derivatives on the Activity of Cellular CK2

Kinase inhibitors which are used as a tool in basic research or as therapeutic agent need to penetrate the cell membrane and affect intracellular CK2. To test whether **4b**, **4a** and **5** are capable to enter the cell, two different cell lines were used: LNCaP cells, an androgen-sensitive prostate carcinoma cell line, and ARPE19, which was derived from a primary culture of retinal epithelium cells [49]. Thus, this represented tumor cells on one hand and normal, non-transformed cells on the other.

**Figure 4:**
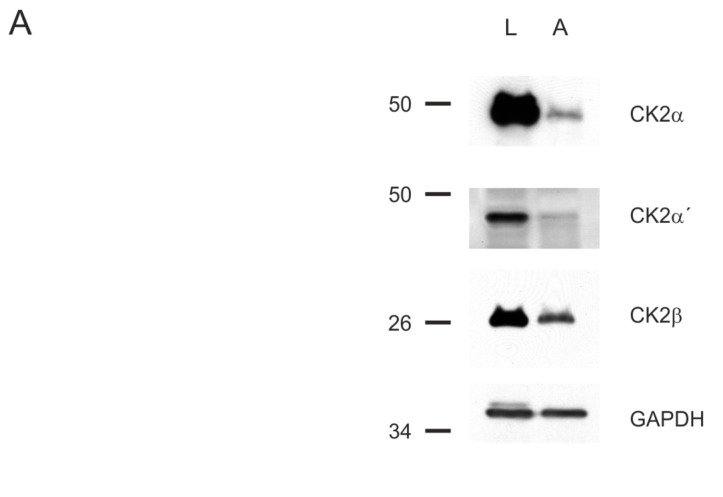

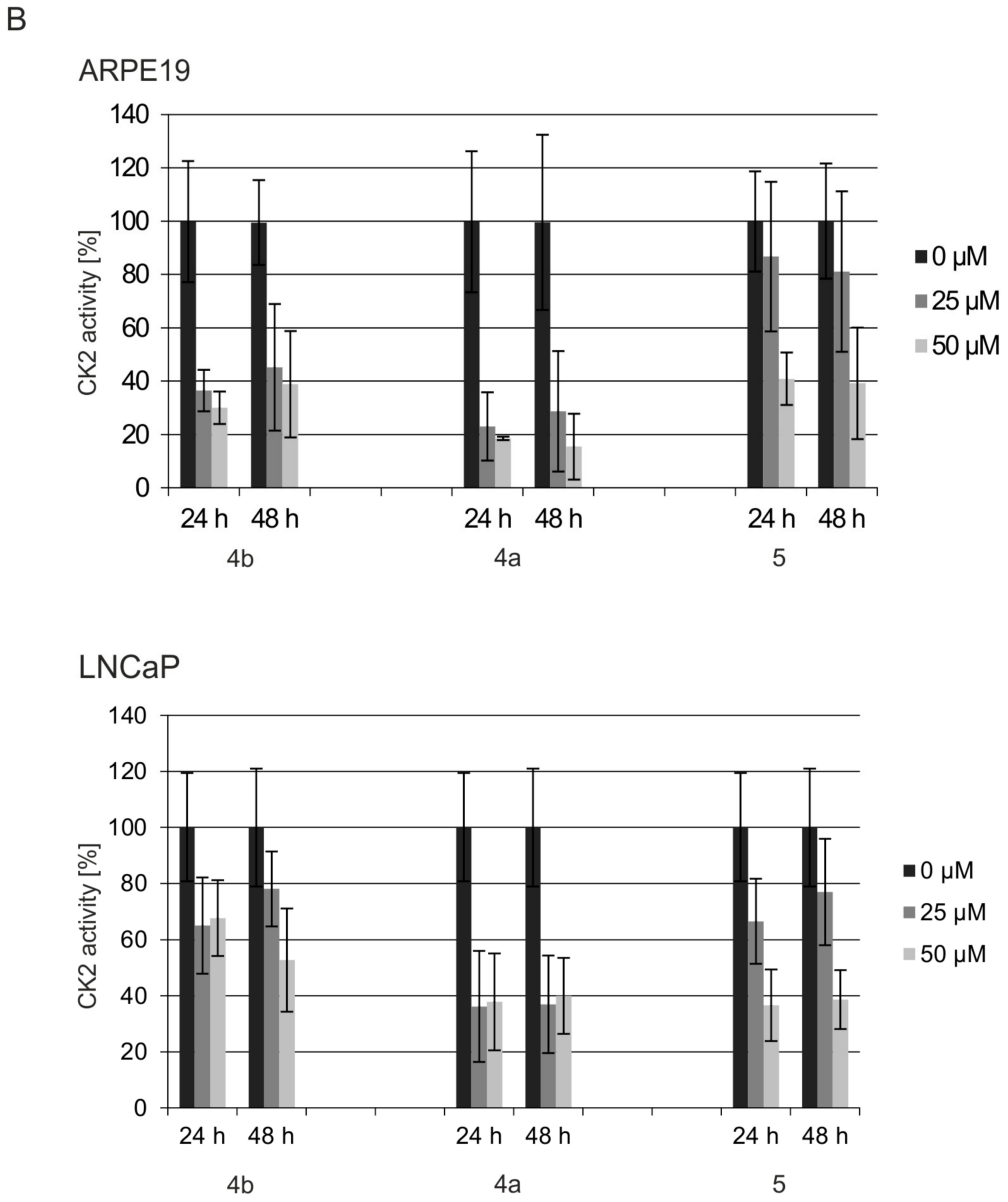
Expression of CK2 in prostate cancer cell line LNCaP and non-neoplastic retinal pigment epithelial cell line ARPE19 and inhibition of intracellular activity by dibenzofurans. (**A**) Cells were extracted with RIPA buffer. 50 μg cell extract was separated on a 12.5% SDS polyacrylamide gel and transferred to a PVDF membrane followed by Western blotting with anti-CK2α (1A5), anti-CK2α’ (#30), anti-CK2α (6D5) and anti-GAPDH antibodies. GAPDH was used as a loading control. (**B**) ARPE19 or LNCaP cells were treated with DMSO as control (0 μM) or with 25 or 50 μM **4b**, **4a** or **5** for 24 or 48 h. Kinase activity of CK2 in the cell extracts was measured with the synthetic CK2 specific peptide substrate, RRRDDDSDDD, in the presence of [[^32^P]γATP. Results from at least three individual experiments are shown; the activity in the control cells was set to 100%.

In a first experiment, it was tested whether CK2 is indeed present in both cell lines. Cells were grown under normal conditions, harvested and the proteins were extracted. After separation by SDS PAGE a western blot analysis was performed with CK2 specific antibodies. The result shown in Figure 4A demonstrated that despite loading fairly equal protein amounts for both cell lines as indicated by the GAPDH loading control, CK2 subunits are much less expressed in the non-neoplastic ARPE19 cells than in the prostate carcinoma cell line LNCaP. This is in agreement with previous observations in different tumor cell lines and tumors and was most obvious for the catalytic α-subunit. In addition a significant difference between both cell lines was observed for the regulatory β-subunit as well. The catalytic α’-subunit developed the weakest signal in both cell lines, and was barely visible in ARPE19 cells. Nevertheless, CK2 was detectable in both cell lines and in subsequent experiments cells were incubated with the dibenzofuran inhibitors followed by an analysis of cellular CK2 activity.

ARPE19 and LNCaP cells were treated with 0, 25 or 50 µM of the different dibenzofuran derivatives **4b**, **4a** and **5** for 24 and 48 h before being harvested. Proteins were extracted and CK2 kinase activity was measured by an *in vitro* phosphorylation assay using the CK2 specific peptide substrate RRRDDDSDDD and radiolabeled [[^32^P]γATP. The activity as measured was normalized to equal protein amounts. The relative CK2 activity of at least three independent experiments is given as bar graphs in Figure 4B. In ARPE19 cells CK2 activity was strongly repressed by all inhibitors. **4b** exerted a 60% inhibition independent of concentration and time. With **4a** an inhibition of 80% was obtained, already by a treatment with 25 µM for 24 h; the application of a higher concentration for a longer time could not improve the inhibitory effect. For **5** a weaker but dose-dependant effect was observed. 25 µM reduced the cellular CK2 activity to 80% and with 50 µM the activity was reduced to 40%. In LNCaP cells the results were essentially similar to those made in ARPE19 cells. However, the repression of activity by **4b** and **4a** was not as strong as in ARPE19 cells. **4b** reduced the activity to 50%, **4a** to 40%. The outcome using **5** was quite similar to the effects observed in ARPE19 cells: a reduction to roughly 80% when using 25 µM, which was enhanced to 40% when using 50 µM. This also indicated cell penetration of compounds **4a**, **4b**, and **5**. A more direct evidence for intracellular inhibition of CK2 could be obtained by measuring the phosphorylation of endogenous natural substrates (biomarker analysis) [50]. This has been done by Guerra and co-workers, who investigated their inhibitor D11, which is identical to our compound **5**, on its cellular effects in different cell lines by measuring inhibition of endogenous substrate phosphorylation [42,51,52]. With respect to their results and taking into consideration that the logP values of compounds **4a**, **4b**, and **5** as given in Table 2 are not of substantial difference, we conclude that the inhibitors were able to cross the cell membrane and exerted a similar inhibitory effect on intracellular CK2 activity in both cell lines.

### 2.5. Induction of Apoptosis and Impact on Tumor Cell Viability

**Figure 5:**
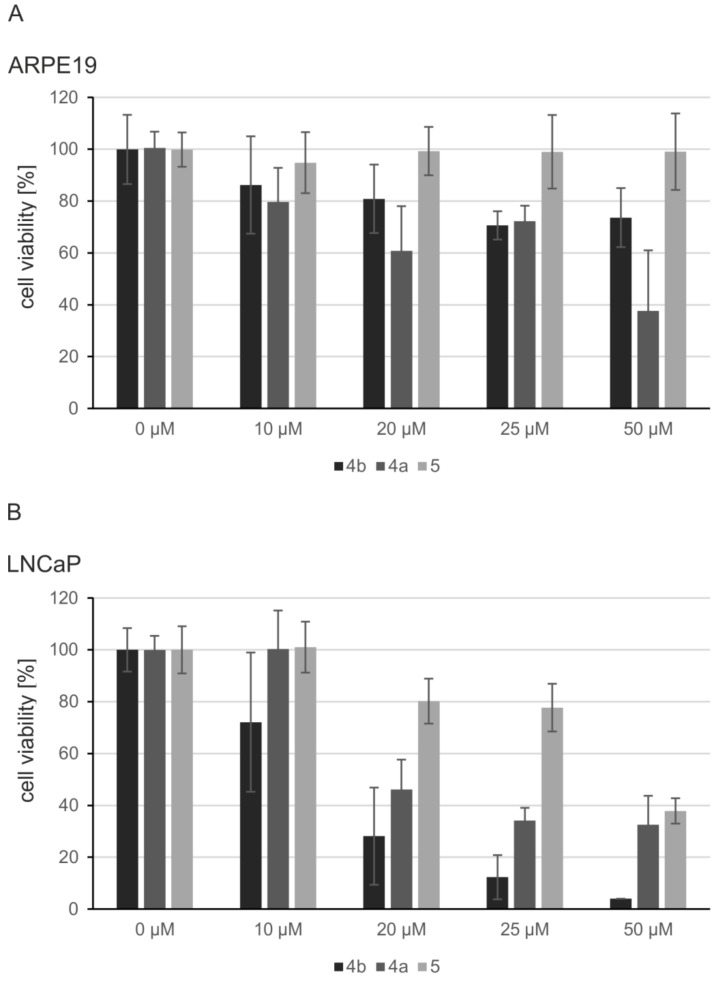
Effects of dibenzofuran inhibitors on cell viability. ARPE19 (**A**) or LNCaP (**B**) cells were incubated with the given concentrations of **4b**, **4a**, **5** or DMSO as control. Cell viability was determined using the MTT assay after 24 h of incubation. Viability was set in reference with DMSO. The mean and standard deviation of three independent experiments with two technical replicates each is shown.

Protein kinase CK2 is essential for the survival of cells. Therefore, we tested the impact of the CK2 inhibitors on the viability of cells in both lines. The inhibitors were applied in increasing concentrations up to 50 µM to growing cell cultures and the viability was analyzed by a MTT assay after 24 h of treatment. The effect of the compounds on the growth of the normal ARPE19 cells was rather moderate (Figure 5A). **5** had no impact on ARPE19 cell viability. **4b** reduced the viability to 70% in its highest concentration and **4a** exerted the strongest influence by reducing the ratio of viable cells to 40% when applying a 50 µM dose. In contrast LNCaP cells were strongly impaired by the treatment with the dibenzofurans (Figure 5B): Using 10 µM of the different inhibitors, we only observed an effect with **4b**. However, increasing the concentration to 20, 25 and 50 µM exerted a drastic impact on the viability of LNCaP cells. With 50 µM **4b** a viability rate of final 5% was obtained, whereas **4a** and **5** reduced the viability to 30 to 35%. Thus, the inhibitors in particular affected the viability of LNCaP carcinoma cells, but not of normal retinal pigment epithelium cells. This observation was supported by the morphology of cells as seen in the microscope (Supplementary Information Figure S3). Even after 48 h of growth under CK2 inhibition ARPE19 cells appeared rather unaffected by the treatment. In contrast, LNCaP cells were severely altered in their morphology by all inhibitors; they could not form a dense cell layer anymore, but detached from the culture dish.

**Figure 6:**
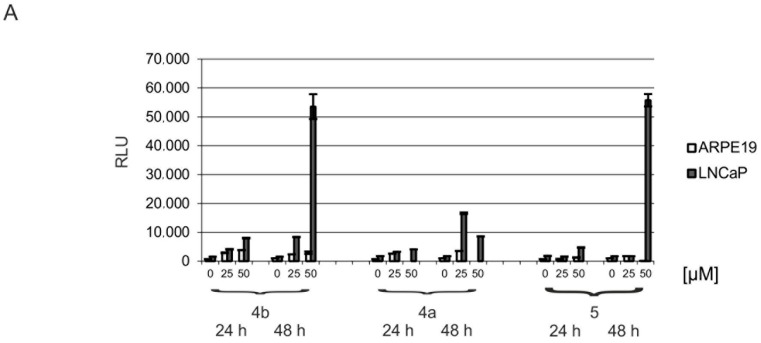

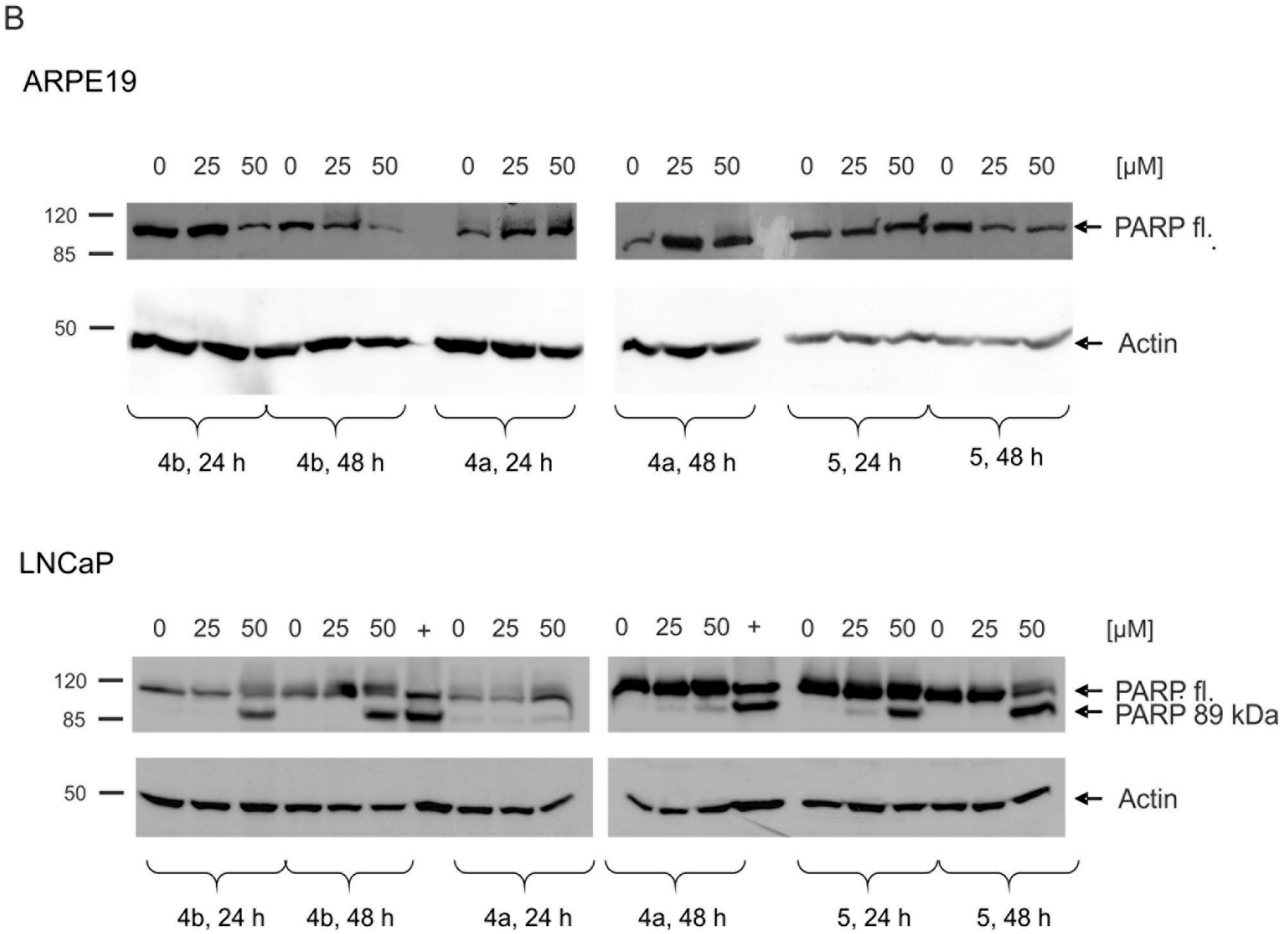
Induction of apoptosis in prostate cancer cell line LNCaP and non-neoplastic retinal pigment epithelial cell line ARPE19. (**A**) ARPE19 or LNCaP cells were treated with DMSO as control (0 μM) or with 25 or 50 μM **4b**, **4a** or **5** for 24 or 48 h. Activity of caspases 3 and 7 was determined using the luminescent Caspase-Glo^®^ 3/7 assay according to the technical bulletin of the manufacturer. Median relative light units (RLU) + standard deviation from at least three independent experiments are shown in the bar graph. (**B**) Cells were treated as described before. 75 μg cell extract was separated in a 10% SDS polyacrylamide gel and analyzed in a Western blot analysis with a rabbit polyclonal antibody against PARP1. + is a positive control for PARP cleavage.

The fact that after 48 h of growth under CK2 inhibition ARPE19 cells were rather unaffected by the treatment whereas in contrast, LNCaP cells were severely constrained by all inhibitors is supporting the concept of the study. As can be seen in Figure 3A, CK2 subunits are strongly overexpressed in LNCaP tumor cells in comparison to ARPE19 cells. Reducing the activity of CK2 by the inhibitors is supposed to overcome the antiapoptotic effect of the enzyme and cells are induced to undergo apoptosis. Whereas in “normal” ARPE19 cells, no apoptotic signals are anticipated, and hence a reduction of CK2 activity should not lead to cell death [53]. In consequence, we analyzed in the next experiments whether the cells were killed by apoptosis. Hallmark of apoptosis is the activation of the key effector caspases 3 and 7 which ends in the cleavage of poly (ADP ribose) polymerase PARP1. The full-length PARP1 is cleaved by the caspases to an 89 and a 24 kDa fragment. These events of late apoptotic signaling were analyzed in ARPE19 and LNCaP cells grown in the presence and in the absence of dibenzofurans. Inhibitor concentrations of 25 and 50 µM were used and were applied for 24 and 48 h. In cell lysates of LNCaP and ARPE19 cells the activity of caspases 3 and 7 was determined subsequently by a luminescence based assay (Figure 6A). In ARPE19 cells only basal caspase activity was observed which never exceeded 4000 RLU (relative luminescence units), regardless of the compound, its applied concentration or the incubation time. However, in LNCaP cells a slight increase of caspase activity was observed already after 24 h, whereas upon treatment with 50 µM of **4b** and **5** after 48 h a significant increase of caspase activity going beyond 50,000 RLU was detectable. With **4a** caspase activity in LNCaP was also enhanced but not as significant as with the two other inhibitors (max. 17,000 RLU in comparison to 55,000 RLU as obtained with **5**). Thus, the activity of the key effector caspases was obviously switched on by 50 µM **4b** and **5** in the tumor cell line LNCaP; **4a** treatment activated the caspases only to a minor degree.

In addition to the luminescence assay, Western blot analysis was performed to check PARP1-cleavage (Figure 6B). Therefore specific antibodies for the detection of the full-length PARP1 (113 kDa) and the larger cleavage fragment of PARP1 (89 kDa) were used. In ARPE19 cells no cleavage product of PARP1 was detectable. In LNCaP we observed the cleavage of PARP1 after treatment with 50 µM **4b** as well as with 50 µM **5**. Treatment with **4a**, however, did not result in cleavage of PARP1.

Thus, we conclude that treatment with all dibenzofuran inhibitors affect the viability of cells, tumor cells being much more sensitive to the treatment than normal cells. The severe drop in viability in tumor cells is obviously due to the induction of apoptosis for **4b** and **5**. **4a** also exerts a cytotoxic effect on LNCaP cells; however, cells do not die by inducing the apoptotic program.

### 2.6. Human CK2α Crystal Structures in Complex with **4a**, **5** and Their Archetype **4b**

In order to elucidate the binding mode of **4b**, **4a** and **5** to CK2 each of the compounds was co-crystallized with *hs*CK2α^1–335^, a fully active C-terminal deletion mutant of *homo sapiens* CK2α [54]. To ensure comparability of the binding modes, care was taken to minimize artifacts of the crystallization media or introduced by crystal packing differences [54]: first we crystallized with polyethylen glycol 4000 (PEG4000) as a precipitant rather than with a kosmotropic salt that might affect potential halogen bonds with aromatic groups [38], second we used in all three cases solutions of comparable compositions for crystallization and for preparation of cryo diffractometry, and third we initiated crystallization by seeding to direct the crystallization process always towards the same crystal packing.

In fact, we obtained isomorphous monoclinic crystals of the three *hs*CK2α^1–335^/inhibitor complexes (Table 3) grown in 32% (*w*/*v*) PEG4000, 0.2 M ammonium acetate, 0.1 M sodium citrate, pH 5.6, i.e., in the presence of moderate salt concentrations. In all three cases well resolved X-ray diffraction data sets were collected which allowed to determine and to refine the underlying complex structures with high quality (Table 3). For the co-crystals with the mother compound **4b** and with its most similar derivative **4a** we collected additional diffraction data with soft X-rays (wavelength 2 Å) in order to localize the chloro substituents of the inhibitors via their anomalous signals [55,56].

**Figure 7:**
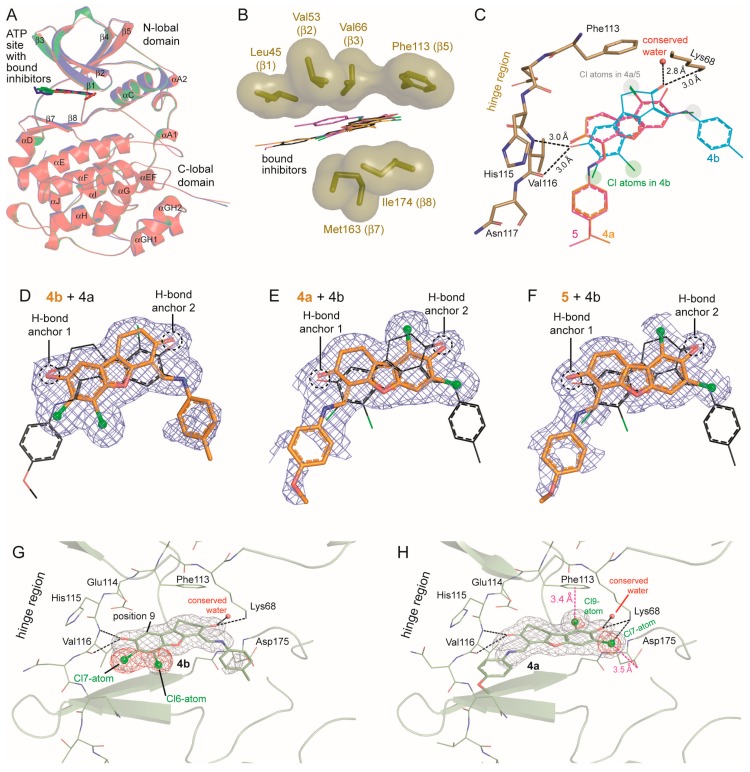
Human CK2α crystal structures in complex with **4b**, **4a**, and **5**. (**A**) Global overview of the three superimposed structures (red: **4b** complex; green: **4a** complex; blue: **5** complex). (**B**) Hydrophobic sandwiching of the dibenzofuran framework by non-polar side chains of the N-lobal strands β1, β2, β3 and β5 from one side and of the C-lobal strands β7 and β8 from the other. (**C**) Each of the inhibitors forms two pairs of hydrogen bonds (drawn in detail for **4b**) with the enzyme. (**D**–**F**) Section of the three inhibitors covered by the final electron densities (cutoff level 0.5 σ) after superimposition of the harboring protein matrices. To facilitate a comparison of the orientations either **4a** (**D**) or **4b** (**E**,**F**) is additionally drawn with black C-atoms. The hydroxy/oxo groups that form hydrogen bonds and that are positionally preserved are indicated by dashed circles. (**G**,**H**) Experimental verification of the different orientations of **4b** and **4a** using the diffraction data sets at 2 Å wavelength (Table 3) at which the anomalous scattering of chlorine is enhanced. In both pictures the respective ligand is covered by its final electron density (grey mesh; cutoff 1 σ). The positions of the chlorine atoms are indicated by red (**G**) anomalous difference Fourier density (cutoff level 4 σ).

As expected, all three protein matrices are structurally largely identical and the three inhibitors bind to the canonical ATP/GTP site of CK2α which is located in a cleft between the two main domains typical for eukaryotic protein kinases (EPKs) (Figure 7A). The three dibenzofuran moieties lie essentially coplanar (Figures 7B,C) apart from the fact the C-atoms 1 and 2 of **4b** and **4a** are sp^3^-hybridized which enforces a certain deviation from planarity in ring C. The dibenzofuran plane is sandwiched by large non-polar side chains located in the β-strands of both main kinase domains (Figure 7B). While this general arrangement is common for binding of ATP-competitive inhibitors in EPKs, there are some CK2α residues like Val66 from the N-lobal side and Ile174 as well as Met163 from the C-lobal domain (Figure 7B) that are conspicuously bulky and hydrophobic in comparison to its equivalents in other EPKs. As a consequence there is a rather narrow hydrophobic cleft with special features like the ability to bind flat and hydrophobic (parts of) molecules in rather diverse ways with respect to peripheral hydrogen and halogen bonds as long as the planar hydrophobic parts are well accommodated. Prime examples of the resulting “freedom in 2D-space” [57] are (i) the dual co-substrate specificity of CK2, i.e., its ability to utilize either ATP or GTP as phospho donor depending on a purine base shift within the binding plane to enable correct hydrogen bonding [58] (ii) the capability of CK2α to bind an inhibitor like emodin without the formation of any hydrogen bond to the peptide backbone of the interdomain hinge which normally provides H-bond anchors for ATP site ligands in protein kinases [55,58] or (iii) the flexibility to switch the halogen bonding pattern by rotations within the binding plane in order to enable the coordination of diverse tetrabromo benzimidazole derivatives [59]. We will show below that **4b** and its derivatives provide a novel and unexpected case of adaptability within CK2α’s purine base binding plane.

Thus, hydrogen bonding to the hinge region of CK2α is in principle not necessary for ATP-competitive CK2 inhibitors. Nevertheless, in order to achieve low-nanomolar K_i_ values the most potent of them like silmitasertib [32] or FLC26 [38] use polar interactions with the hinge and, in addition, with a hidden region next to Lys68 known for its positive electrostatic potential and for the presence of conserved water molecules [60]. Consistent with this rule, each of the tight binding inhibitors **4b**, **4a** and **5** forms four hydrogen bonds with *hs*CK2α^1–335^ (Figure 7C). For this purpose they exploit the hydrogen bonding potential of the two oxo or hydroxy substituents at positions 3 and 8 (Figure 1). Each of them is the origin for one pair of hydrogen bonds (Figure 3C) either with the peptide groups of Val116 in the hinge region or with the side chain of Lys68 and a neighboring, highly conserved water molecule [60]. The enzyme is pre-formed for these hydrogen bonds and requires no structural adaptations: in the human CK2α apo-structure 3WAR which is the highest resolved CK2α structure published so far [61] the H-bond partners on the protein side are essentially at the same position as in the *hs*CK2α^1–335^ complexes with **4b**, **4a** and **5**.

Remarkably, on the inhibitor side the two central H-bond positions are occupied in all three cases by suitable oxygen substituents (H-bond anchors 1 and 2 in Figure 7D–F), irrespective of the fact that—as illustrated in Figures 7D–F and as described below in more detail—the overall orientation of **4b** is completely different from that of **4a** and **5**. A further note deserves the fact that Val116 is in one of its two H-bonds the hydrogen donor, but in the other one the hydrogen acceptor: this is only possible if a hydroxy rather than an oxo group is the H-bond partner at the inhibitor side. This condition is directly matched for **4b** and for **5** where the OH-group at position 8 (**4b**) or at position 3 (**5**) is the common H-bond anchor for Val116. In contrast, in **4a** the substituent at position 3 which is located at the equivalent position is formally an oxo group (Figure 1), which suggests that keto-enol tautomerization must have occurred in **4a**.

While the three-ring systems of the inhibitors are well defined by electron densities this is not the case with the distal methylphenyl or methoxyphenyl groups: only if the contouring level is lowered to 0.5 σ, are faint and disrupted pieces of electron density visible for these parts of the molecules (Figures 7D–F). As we do not have any experimental evidence for a general instability of the connecting linker, the observation may be the consequence of configurational and conformational flexibilities in this region resulting from the keto-enol tautomerization. In any case, these flexibilities are preserved in the enzyme-bound states and obviously not disadvantageous for the affinities.

In spite of the fact that each of the inhibitors contains two candidate chloro substituents neither of them forms a halogen bond to one of the peptide carbonyl oxygens at the hinge region as it is often found for protein kinase inhibition by halogen-containing ATP-competitive ligands [62]. Quite the opposite, the two chlorine atoms of **4b** at positions 6 and 7 (Figure 1) whose locations are clearly confirmed by anomalous difference density peaks (Figure 7G) point away from the hinge region backbone; they are not halogen-bonded at all resembling in this respect the recently described binding mode of the flavonol derivative FLC26 [38]. Intuitively one would expect that a relocation of **4b**’s Cl6-atom to position 9 (Figure 7G) as it is in **4a** and in **5** would bring the chlorine atom into the proximity of the hinge residue Glu114 and should therefore generate a Cl-O halogen bond with the peptide oxygen of Glu114. Surprisingly, however, neither **4a** nor **5** forms such a halogen bond to the hinge backbone; rather, in their complexes with *hs*CK2α^1–335^ the entire dibenzofuran scaffold is rotated compared to **4b**, namely by 180 degrees around the pseudo-twofold symmetry axis running in-plane through the furan ring (ring B in Figure 1) followed by a 38 degree rotation perpendicular to this axis (Figure 7D–F). To validate this reversal of the orientation we calculated an anomalous difference density in the case of **4a**; it unambiguously shows the two chloro substituents lying in the vicinity of Phe113, Lys68 and Asp175 (Figure 7H), i.e., deeply inside the ATP/GTP cavity, but far away from the hinge region. To our knowledge, no other CK2α structure with a halogenated inhibitor exists with a comparable location of the halogen atoms.

Thus, rather than using their novel Cl9-atom to form a Cl-O halogen bond at the hinge region, **4a** and **5** prefer to establish a π-halogen bond with the phenyl ring of Phe113 as indicated in Figure 7H. In both complexes the distance of Cl9-atom to the centroid of the phenyl ring is 3.4 Å which is fairly close compared with average values of 3.6 Å or 3.854 Å reported by Matter et al. [63] and Lu et al. [64] In addition to this vigorous halogen bond, the nearby Cl7 atom is hydrogen bonded to the Lys68 side chain and in halogen-bond distance (3.5 Å) to one of the oxygen atoms to the side chain of Asp175.

In summary, in complex with *hs*CK2α^1–335^ all three inhibitors make very similar hydrophobic interactions and hydrogen bonds with the enzyme. However, with respect to halogen bonds **4a** and **5** differ significantly from **4b** since they fit so perfectly to the structural environment provided by the enzyme that a strong novel π-halogen bond is established which reduces their affinity to the subnanomolar range. This finding resembles an observation of Matter et al. [63] who reported about the optimization of two protease inhibitors in which the switch of an aromatic chloro substituent from the *meta-* to the *para*-position led to a drop of K_i_ values by about two orders of magnitude and who presented evidence that this behavior is due to π-halogen bonds.

## 3. Discussion

A first outcome from this study is the confirmation that dibenzofurans are potent lead structures for human protein kinase CK2 inhibitors [37]. This is additionally supported by recent studies of Guerra et al. [42], who identified dibenzofuran “D11”, which appears to be identical to **5** by screening of 1.600 compounds from the NIH/NCI Diversity Set on CK2 inhibition. However, they did neither describe the synthesis of the compound nor clarify the structural basis of its binding to CK2. D11 turned out to be able to induce caspase-mediated cell death in human glioblastoma and pancreatic adenocarcinoma cells lines, which have been shown before to be resistant to conventional chemotherapeutic agents [50]. Finally, first indications on the influence on intracellular signaling and on the phosphoproteome by D11 treatment were obtained [50,51]. In this study, cell permeability of **4a** and **5** could be shown as well, in particular by the strong inhibition of intracellular CK2 activity in the prostate cancer cell line LNCaP and in the non-neoplastic retinal pigment epithelial cell line ARPE-19. To our surprise, **5** showed no significantly weaker effect on intracellular CK2 activity, despite an unfavorable high logP value of 6.02 (Table 2) in comparison to **4a** (4.55) and **4b** (4.95), the mother compounds of both new structures [28,37]. Moreover, **4b** reduced cell viability of prostate cancer cell line LNCaP with a significant stronger degree than **4a** and **5**, which exhibited a similar effect, despite their quite different logP values. Here we need to emphasize, that in comparison to **4a** (IC_50_ = 7 nM) and **5** (5 nM), **4b** (15 nM) was the weakest inhibitor of CK2. This indicates that additional effects beyond inhibition of CK2 contribute to the observed differences in cellular activity. A first reason could be the different cell permeability of the compounds as indicated by their different logP values and TSPA values (Table 2), but this needs further experimental conformation, e.g., by a Caco-2 assay, as active transport into the cells or even out of the cells again by multi-drug resistance proteins is not considered by values as logP or TSPA. In addition, it cannot be excluded that multivalent effects, i.e., interactions of the compounds with other cellular targets than CK2 contributed to the cellular effects. In this context, it appears remarkable, that despite being structurally rather similar, the three compounds analyzed, **4b**, **4a** and **5** showed a different selectivity pattern, when tested against a panel of 61 human protein kinases (Table 1 and Supplementary Figure S2). The only two kinases beyond CK2 that were inhibited to more than 70% by all of the three compounds to more than 70% were Aurora 1 and SKG1. Both, **4a** and **5** additionally inhibited CAMKK2, DYRKB1, FLT3 and the D835Y mutant of FLT3 to a similar extent. In contrast, **4b** as well as **5** inhibited at 10 µM concentration FLT4/VEGRF3, KDR/VEGF2, PIM1 and LCK to almost 70% or more. This is surprising because from a structural point of view, **4b** seems to be more similar to **4a** than to **5** (Figure 1). PKA and C-met was only inhibited by **5** to more than 70% at 10 µM, and PKD/PRKD2 was only inhibited by **4b** at the same concentration to a similar extent. Although these results need to be confirmed and must be considered as preliminary, because only one concentration of each inhibitor was tested in the presence of one concentration of ATP, independent of the different K_M_ values for the co-substrate with each kinase, such a heterogeneous pattern as observed was not to expect. It may be an indication, that indeed multi-target effects could have contributed to the difference in cellular effects of the three compounds. In addition it emphasizes, that multi-target effects, also named as polypharmacology more recently [65,66], are worth to be considered at early stages of drug discovery and could be subjected to a rational approach. Hence, an observed lack in selectivity must not be a flaw, but could even be beneficial if the compound addresses multiple targets related to the same disease and the interaction can be measured and as well be controlled by distinct structural features. First polypharmacology approaches of CK2 inhibitors with the aim to identify the structural requirements for inhibiting additional targets such as CDC25 and ABCG2 have been published recently [67,68]. Although we are still at beginning of the rational design of multi-target drugs, the understanding that addressing multiple targets could be beneficial for therapeutic intervention (and as such reflecting the multifactorial genesis of disease and re-echoing the observation of a poor correlation between *in vitro* drug effects and *in vivo* efficacy) will lead to completely new strategies in drug discovery.

In summary, both new compounds, **4a** and **5** turned out to be tight binding inhibitors of human CK2 with IC_50_ values of 5 nM (**4a**) and 7 nM (**5**) and an apparent K_i_-value of 0.4 nM (**4a**, **5**). Schaefer et al. [51] investigated D11, which is structurally identical to our compound **5** on a possible binding mode. They supposed a Michaelis-Menten kinetics and tried to determine the K_i_ value of D11 by increasing concentrations of the co-substrate ATP with three different concentrations of inhibitor. The outcome was somewhat intriguing, as this resulted in a decreased apparent maximum velocity (V_app_ max) and increased apparent K_M_ (K_Mapp_) values. From these results the authors concluded a linear mixed-type inhibition. Because the enzyme concentration used in these assays was not specified, it is difficult to evaluate, that indeed “steady state” conditions were maintained and a Michaelis-Menten kinetics can indeed be postulated. In the light of our results—the inhibition kinetics shown in Figure 3 and the structural results demonstrating that the inhibitor binds to the ATP-site of CK2α and nowhere else—we conclude, that the results obtained with D11 by Schaefer et al. [51] on the kinetics of inhibition are also reflecting “tight-binding” mode.

Compounds 4a and 5 showed a similar level of selectivity as 4b, and also induced apoptosis in the prostate cancer cell line LNCaP, but not in ARPE-19, a non-tumor derived human retinal pigment epithelial cell line. Co-crystal structure determination with the three compounds and *hs*CK2α^1–335^ [69] revealed, that changing the chloro-substituent from the 6 position of the dibenzofuran scaffold—as in 4b—to the 9 position as in 4a and 5 resulted in the formation of a π-halogen bond with the side chain of the “gatekeeper” residue Phe113. Without precedent, the formation of this π-halogen bond led to a complete inversion of the binding mode in comparison to 4b, where the non-halogenated phenyl-ring is a stacking partner of Phe113. To our knowledge, this is the first example for inverting the binding mode of an inhibitor by moving the position of a chloro-substituent without reducing its potency. It emphasizes the importance of halogen bonds—weak noncovalent interaction with electron rich moieties and the so-called σ-hole in line with the covalent C-halogen bond [40]—within the context of ligand/target binding, and it will redirect the view on the role of aromatic residues for interaction with halogen containing compounds in drug discovery. The synthesis strategy as applied here and which led to the discovery of 4b, 4a and 5, is limited concerning the pattern of chloro substituents at ring A and the investigation of homologues compounds without a ring D. It is based on the reaction of dichloro-*p*-benzoquinones with 2-[(arylamino)methylene]cyclohexanone derivatives to yield the dibenzofurans as described here [70]. Therefore a new synthesis strategy is required in order to systematically analyze the influence of the position of the chloro substituents at ring A for the orientation of the dibenzofurans in the ATP pocket and inhibitor potency. It will also be needed for elucidating the contribution of ring D to the inhibitory effect and which part the flexible or rigid connection to the dibenzofuran moiety plays in this context. This is under current investigation with the aim to perform QSAR studies in a more systematic approach with variants of the dibenzofuran core.

## 4. Materials and Methods

### 4.1. General Information

Spectra of the compounds were determined in a Genesys 10SUV/Vis spectrophotometer (Thermo Scientific, Waltham, MA, USA). Purity of the comopunds was determined by HPLC (Elite LaChrom, VWR Hitachi, Langenfeld, Germany), combining a RP18 column with a diode arry detector. The enzymatic activity of CK2 with and without inhibitors was quantified in a CE PA800 plus (Beckman-Coulter, Krefeld, Germany). For recording of NMR spectra, a Varian AS Mercuryplus NMR spectrometer (Agilent, Santa Clara, CA, USA) was used and an IR Affinity-1 (Shimadzu, Kyoto, Japan) for IR spectra. Human cell lines were cultivated in a cell culture incubator Heracell 240i (Thermo Scientific, Waltham, MA, USA) and microscoped by a Zeiss Axiovert 25 (Zeiss, Jena, Germany). Proteins were seperated by SDS-PAGE in a Mini-Protean Tetra System and for Western blotting a Western Blot Miniprotean II cell (both from Bio-Rad, München, Germany) was used.

### 4.2. Chemistry

The synthesis of 7,9-dichloro-1,2-dihydro-8-hydroxy-4-[(4-methoxyphenylamino)methylene]dibenzo[*b*,*d*]furan-3(2*H*)-one (**4a**), 6,7-dichloro-1,4-dihydro-8-hydroxy-4-[(4-methylphenylamino)methylene]dibenzo[*b*,*d*]furan-3(2*H*)-one (**4b**), and (*E*)-1,3-dichloro-6-[(4-methoxyphenylimino)methyl]dibenzo[*b*,*d*]furan-2,7-diol (**5**) has been described before [37,39]. For better understanding, a brief summary of the synthesis steps and analytics is given.

#### 4.2.1. Synthesis of 7,9-dichloro-1,2-dihydro-8-hydroxy-4-[(4-methoxyphenylamino)methylene]dibenzo[*b*,*d*]furan-3(2*H*)-one (**4a**).

2,6-Dichloro-1,4-benzoquinone (1.77 g, 0.01 mol) and 2-[(4-methoxyphenylamino)methylene]cyclohexanone (4.62 g, 0.02 mol) were heated under reflux in dichloromethane (50 mL) for 60 min to yield a green coloured solution. After cooling to room temperature 2,6-dichloro-1,4-benzoquinone (3.54 g, 0.02 mol) dissolved in glacial acetic acid (10 mL), followed by refluxing again for 30 min. Filtration of the precipitate formed after standing overnight yielded 1.2 g (14.9%) of a pure orange product (mp 204 °C). IR (KBr) 1665 cm^−1^. UV (CH_2_Cl_2_) λ_max_ 406 nm (lg ε = 4.35). ^1^H-NMR (pyridine-d_5_) δ 2.80 (mc, 2H, CH2); 3.20 (mc, 2H, CH2); (DMSO-d_6_): 3.75 (s, 3H, OCH3); 7.00 (mc, 2H, arom. H) and 7.20 (mc, 2H, arom. H); 7.42 (s, 1H, H-6); 7.74 (d, J = 12.0 Hz, 1H, CH=N); 9.56 (s,1H, OH); 11,56 (d, J = 12.0 Hz, 1H, NH). ^13^C-NMR (DMSO-d_6_): δ 17.8 (t, C-1), 37.0 (t, C-2), 197,2 (s, C-3), 104.5 (s, C-4), 145.2 (s, C-4a), 147.0 (s, C-5a), 110.2 (d, C-6), 111.7 (s, C-7), 156.2 (s, C-8), 116.7 (s, C-9), 126.1 (s, C-9a), 99.4 (s, C-9b), 137.4 (d, C-10), 133.0 (s, C-1”), 118.4 (d, C-2”/C-6”), 114.8 (d, C-3”/C-5”), 154.3 (s, C-4”), 55.3 (q, OCH3). C_20_, H_15_Cl_2_NO_4_ (404.3). C, 59.42; H, 3.74; Cl, 17.54; N, 3.46. Found: C, 59.50; H, 3.72; Cl, 17.49; N, 3.28. Purity was determined to be >95%.

#### 4.2.2. Synthesis of 6,7-dichloro-1,4-dihydro-8-hydroxy-4-[(4-methylphenylamino)methylene]-dibenzo[*b*,*d*]furan-3(2*H*)-one (**4b**)

2,6-Dichloro-1,4-benzoquinone (1.77 g, 0.01 mol) was mixed with 2-[(4-methylphenylamino)methylene]cyclohexanone (4.30 g, 0.02 mol) in dichloromethane (50 mL) and heated under reflux for 60 min to yield a green coloured solution. After cooling to room temperature, 2,6-dichloro-1,4-benzoquinone (3.54 g, 0.02 mol) dissolved in glacial acetic acid (10 mL) was added followed by refluxing again for 30 min. After standing overnight, filtration of the precipitate yielded 1.55 g (20%) of pure product (mp 263 °C). Yellow needles (Ethanol). IR (KBr) 1650 cm^−1^. ^1^H-NMR (pyridine-d_5_) δ 2.20 (s, 3H, CH3), 2.73 (m, 4H, CH2-CH2), 7.17 (m, 5H, arom. H), 8.00 (d, J = 12.0 Hz, 1H, CH=), 8.73 (s, 1H, OH), 11.93 (d, J = 12.0 Hz, 1H, NH). C_20_H_15_Cl_2_NO_3_ (388.25): C, 61.87; H, 3.89; Cl, 18.26; N, 3.61. Found C, 61.72; H, 3.88; Cl, 18.39; N, 3.50. Purity was >95%.

#### 4.2.3. Synthesis of (*E*)-1,3-dichloro-6-[(4-methoxyphenylimino)methyl]dibenzo[*b*,*d*]furan- 2,7-diol (**5**)

2-[(4-Methoxyphenylamino)methylene]cyclohexanone (0.81 g, 2.0 mmol) and 2,3-dichloro-5,6-dicyano-1,4-benzoquinone (0.5 g, 2.2 mmol) were suspended in dichloromethane (50 mL), glacial acetic acid (30 mL) was added and the mixture was refluxed for 10 min. After cooling the red precipitate was filtered and recrystallized from toluene to yield 0.56 g (70%). Mp 210 °C. IR (KBr) 1625 cm^−1^. C_20_H_13_Cl_2_NO_4_ (402.2). ^1^H-NMR (DMSO-d_6_) δ 3.83 (3H, s, OCH_3_), 6.95 (1H, d, J = 8 Hz, 8-H), 7.0–7.6 (5H, m, arom. H), 7.73 (1H, s, H-C=N-), 8.14 (1H, d, J = 8 Hz, 9-H), 9.16 (1H, s, OH). C, 59.72; H, 3.26; Cl, 17.63; N, 3.48. Found C, 59.49; H, 3.10; Cl, 18.00; N, 3.64. Purity was determined to be >95%.

### 4.3. CK2 Inhibition Assay and Kinetic Determinations

In order to determine the inhibition toward human protein kinase CK2 holoenzyme, the dibenzo[*b*,*d*]furan derivatives were subjected to the CE-based CK2 assay [71], as already described for other novel CK2 inhibitors before [70]. In summary, 78 µL of kinase buffer (50 mM Tris/HCl, pH 7.5, 100 mM NaCl, 10 mM MgCl_2_ and 1 mM DTT) containing 0.25 µg CK2 holoenzyme were supplemented with 2 µL of the test-compound dissolved in DMSO and incubated at 37 °C for 10 min. After the addition of 120 µL of assay buffer (25 mM Tris/HCl, pH 8.5, 150 mM NaCl, 5 mM MgCl_2_, 1 mM DTT, 190 µM substrate peptide RRRDDDSDDD and 100 µM ATP) the CK2 reactions were carried out for 15 min at 37 °C, before they were stopped by ice cooling and the addition of 5 µL of EDTA (10 mM, pH 8.0). The samples were then analyzed for phosphorylation of the CK2 substrate peptide by capillary electrophoresis. For the IC_50_-determinations, 13 different concentrations of the test compounds ranging from 0.01 nM to 10 µM were subjected to the test. Controls for 100% and 0% inhibition were realized by samples containing pure DMSO and samples additionally lacking the CK2 holoenzyme. IC_50_ were calculated from the obtained dose response curves.

The three dibenzo[*b*,*d*]furan derivatives **4b**, **4a** and **5** were tested on selective inhibition toward one of the two isoform CK2 holoenzymes (CK2α_2_β_2_ and CK2α’_2_β_2_). For this purpose, their IC_50_ were determined following the protocol of the recently described whole-cell CK2 selectivity assay [47]. In summary, 78 µL of a bacterial suspension of *Escherichia coli* BL21 (DE3) either surface displaying CK2α + CK2β or CK2α’ + CK2β in kinase buffer (50 mM Tris/HCl, pH 8.5, 100 mM NaCl, 10 mM MgCl_2_ and 1 mM DTT) were supplemented with 2 µL of the test compound dissolved in DMSO and incubated at 37 °C and 400 rpm for 10 min. CK2 reactions were started by the addition of 120 µL of assay buffer (25 mM Tris/HCl, pH 7.5, 150 mM NaCl, 5 mM MgCl_2_, 1 mM DTT, 167 µM substrate peptide RRRDDDSDDD and 1 mM ATP) leading to a total reaction volume of 200 µL with final concentrations of 100 µM for the substrate peptide and 600 µM for ATP. The final cell densities had an OD578 of 2 (corresponds to ≈1.7 × 108 cells/mL) [48]. After the samples were incubated for 120 min at 37 °C and 400 rpm, the CK2 presenting bacteria were removed by centrifugation (3850× *g*, 5 min, 4 °C). The obtained supernatants were transferred to the wells of a 96 well-microplate and finally supplemented with EDTA to a concentration of 12.5 mM to definitely stop the enzymatic reactions. The samples were then subjected to a Beckman Coulter pa800 plus (Krefeld, Germany) CE system and analyzed for phosphorylation of the substrate peptide. Samples containing pure DMSO served as controls for 0% inhibition and samples additionally lacking ATP served as controls for 100% inhibition. Nine different concentrations of the compounds ranging from 1 nM to 100 µM in case of **4b** and 0.1 nM to 10 µM in case of **4a** and **5** were applied to the test. IC_50_ values were calculated from the resulting dose-response curves.

### 4.4. Cell Culture and Treatment of Cells with Dibenzofuran Derivatives

ARPE19 cells (ATCC Number: CRL-2302), retinal pigment epithelia (RPE) cells, were maintained at 37 °C and 5% CO_2_ in Dulbecco’s modified Eagles medium (DMEM) supplemented with 2 mM l-glutamine, 1 mM gentamicin and 10% fetal calf serum (FCS) at 37 °C in an atmosphere enriched with 5% CO_2_. The hormone-sensitive prostate cancer cell line LNCaP (ATCC: CRL-1740, passage number 30–40) was cultured in RPMI1640 (Sigma-Aldrich Chemie GmbH, Munich, Germany) supplemented with 10% fetal bovine serum and 2 mM l-glutamine at 37 °C in an atmosphere enriched with 5% CO_2_. 4b, 4a and 5 were dissolved in DMSO as a 10 mM stock solution and cells were treated in the concentrations and times as indicated. Pure DMSO was applied as control. For the evaluation of cell morphology ARPE19 and LNCaP cells were analysed with bright field microscopy using a Axiovert microscope from Zeiss (Jena, Germany).

### 4.5. MTT Viability Assay

Cell proliferation and viability was determined using a colorimetric MTT-based assay (MTT: 3-[4,5-dimethylthiazol-2-yl]-2,5-diphenyl tetrazolium bromide, Sigma-Aldrich, Deisenhofen, Germany) as described in [72]. The assay is based on the activity of mitochondrial dehydrogenases.

### 4.6. Extraction of Proteins

For harvesting cells—including floating cells—were scraped off the plate with a rubber policeman and sedimented by centrifugation (7 min, 4 °C, 400× *g*). Cells were washed with cold phosphate buffered saline (PBS) and the cell pellet was lysed with the double volume of RIPA buffer (50 mM Tris/HCl, pH 8.0, 150 mM NaCl, 0.5% sodium desoxycholate, 1% Triton X-100, 0.1% sodium dodecylsulfate) supplemented with the protease inhibitor cocktail completeTM (Roche Diagnostics GmbH, Mannheim, Germany). After lysis cell debris was removed by centrifugation. The protein content was determined according to a modified Bradford method (BioRad, Munich, Germany).

### 4.7. Determination of Cellular CK2 Activity by an In Vitro Phosphorylation Assay

To determine the activity of CK2 after application of the inhibitor, cells were treated with or without **4b**, **4a** or **5**, lysed and the extracts used in a kinase filter assay as described by Schwind et al. [73].

### 4.8. Western Blot Analysis

Proteins were separated by SDS polyacrylamide gel electrophoresis and blotted onto a PVDF membrane. After blocking (1 h with PBS-Tween20 with 5% dry milk) the membrane was incubated with appropriate dilutions of primary antibodies in PBS-Tween20 with 1% dry milk for another hour at room temperature or at 4 °C overnight. Then the membrane was washed twice for 10 min with PBS-Tween20 with 1% dry milk. Incubation with the peroxidase-coupled secondary antibody (anti-rabbit 1:30,000, anti-mouse 1:10,000) followed for 1 h. The membrane was washed again twice for 10 min with PBS-Tween20. Signals were developed and visualized by the Lumilight system of Roche Diagnostics GmbH. For the detection of the apoptose marker protein PARP we used a polyclonal PARP antibody (Cell Signaling Technology, Frankfurt, Germany), a GAPDH specific antibody (Santa Cruz Biotechnologies, Dallas, TX, USA) was used for loading control. For the detection of the different CK2 subunits we used the mouse monoclonal antibody 1A5 for the catalytic α-subunit [74], the mouse monoclonal antibody 6D5 for the regulatory β-subunit [75] and the rabbit anti-peptide serum #30 for the catalytic α’-subunit [76].

### 4.9. Assay of Caspase Activity

The activity of the apoptotic key effector caspases 3 and 7 in cell extracts was determined using the Caspase-Glo 3/7 assay according to the protocol of the manufacturer (Promega, Mannheim, Germany).

### 4.10. Crystallization and Structure Determination

Each of the three inhibitors 4b, 4a and 5 were separately crystallized with *hs*CK2α^1–335^. Prior to the crystallization these inhibitors were solubilized in 100% DMSO in a concentration of 10 mM. 4a and 5 were mixed with *hs*CK2α^1–335^ (8–10 mg/mL in 500 mM sodium chloride, 25 mM Tris/HCl pH 8.5) in a ratio of 1:10. 4b was mixed in a ratio of 1:5. After a short time of incubation, these mixtures were merged with reservoir solution (32% (*w*/*v*) PEG4000, 0.2 M ammonium acetate, 0.1 M citrate pH 5.6) in a ratio of 2.5:1. 3.5 µL of these mixtures were then equilibrated against the reservoir solution (4b and 4a: 1 mL; 5: 100 µL). The crystal growth was induced by seeding with 150 nL seeding solution after an equilibration time of two days. Grown crystals were harvested after one week.

The crystals obtained in this way were cryo-protected by incubation in a vitrification solution [32% (*w*/*v*) PEG4000, 0.2 M ammonium acetate, 5% (*v*/*v*) glycerol, 0.1 M citrate pH 5.6, 2 mM of the co crystallized inhibitor] for 1 min followed by vitrification in liquid nitrogen.

The subsequent X-ray diffractometry experiments were performed at beamline ID23-2 of the European Synchrotron Radiation Facility (ESRF) in Grenoble, France, and at beamline X06DA (PXIII) of the Swiss Light Source (SLS) in Villigen, Switzerland. We collected high resolution data from each of the *hs*CK2α^1–335^/inhibitor crystals (Table 3); in the case of the *hs*CK2α^1–335^ complexes with **4b** and **4a** we afterwards used the same crystal, respectively, to collect a “soft” X-ray diffraction data set (wavelength 2 Å) in order to unambiguously validate the positions of the inhibitors’ Cl-substituents via their contribution to anomalous diffraction. PDB codes: **4a**: 5N9N; **4b**: 5N9L; **5**: 5N9K.

All X-ray diffraction data were integrated with XDS [77] followed by POINTLESS [78] for symmetry determination, AIMLESS [78] for scaling and CTRUNCATE as well as some other programs of the CCP4 software suite [79] for the final steps of data reduction to structure factor amplitudes.

The structures were solved by molecular replacement with PHASER [80] using the PDB file 2PVR [81] as a template. For refinement we used PHENIX [82], manual modelling was made with Coot [83]. The topology of the three inhibitors were constructed with the help of PRODRG [84]. The anomalous difference density maps for the *hs*CK2α^1–335^ complexes with **4b** and **4a** were generated with the corresponding map calculation routine of PHENIX [82].

## Figures and Tables

**Table 1:** Selectivity profile of dibenzofuran derivatives: human kinases that were inhibited more than 70% at 10 µM concentration.

4a	5	4b
Aurora A	Aurora A	Aurora A
SKG1	SKG1	SKG1
CAMKK2	CAMKK2	
DYRKB1	DYRKB1	
FLT3 (D835Y)	FLT3 (D835Y)	
FLT3	FLT3	
	FLT4/VEGFR3	FLT4/VEGFR3
	KDR/VEGFR2	KDR/VEGFR2
	PIM1	PIM1
	LCK (68%) *	LCK
	PKA	
	C-Met	
		PKD/PRKD2

* At 10 µM concentration inhibition was 68%.

**Table 2:** TPSA and logP values of dibenzo[*b*,*d*]furan derivatives.

Compound	logP	TPSA (A^2^)
4b	4.95	62.47
4a	4.55	71.70
5	6.02	75.19

**Table 3:** X-ray diffraction data collection and refinement statistics.

Complex	*hs*CK2α^1–335^/4b		*hs*CK2α^1–335^/4a		*hs*CK2α^1–335^/5
X-ray diffraction data collection					
Wavelength [Å]	1.0	2.0	1.0	2.0	1.0
Synchrotron (beamline)	SLS (PX-III)				
Space group	P2_1_				
Lattice constants					
a, b, c [Å]	57.44, 45.71, 63.19	57.43, 45.38, 63.33	57.48, 45.51, 63.49	57.67, 45.50, 63.32	57.88, 45.67, 63.75
α, β, γ [°]	90, 110.94, 90	90, 110.81, 90	90, 110.97, 90	90, 110.81, 90	90, 111.14, 90
Protomers per asymmetric unit	1	1	1	1	1
Resolution [Å] (highest res. shell)	34.79–1.79 (1.85–1.79)^1^	36.01–2.90 (3.00–2.90)	34.16–1.84 (1.91–1.84)	36.10–2.99 (3.10–2.99)	36.22–1.64 (1.70–1.64)
R_sym_ [%]	5.4 (51.3)	3.3 (6.1)	5.5 (70.6)	9.2 (28.6)	3.5 (61.5)
CC1/2	0.998 (0.818)	0.999 (0.998)	0.999 (0.705)	0.996 (0.958)	0.999 (0.738)
Signal-to-noise ratio (I/σ_I_)	12.86 (1.74)	43.5 (26.8)	14.02 (1.55)	16.00 (5.20)	18.71 (1.70)
No. of unique reflections	27,735 (2290)	6609 (610)	25,781 (2428)	6058 (525)	37,646 (3505)
Completeness [%]	95.24 (79.14)	95.3 (88.2)	96.10 (90.42)	94.4 (84.2)	98.85 (93.39)
Multiplicity	3.5 (3.1)	6.5 (6.8)	3.5 (3.2)	6.5 (6.1)	3.3 (3.0)
Wilson B-factor [Å^2^]	25.37	29.55	28.71	48.50	23.85
Structure refinement with high-resolution data sets					
No. of reflections for R_work_/R_free_	26,336/1389		14,456/1317		36,809/1826
R_work_/R_free_ [%]	16.35/19.92		16.70/20.05		16.38/18.43
Mean coordinate error [Å]	0.180		0.190		0.180
No. of non-H-atoms	3094		3055		3147
Protein	2818		2803		2841
Ligands/ions	58		77		57
Water	218		175		249
Mean B-factors [Å^2^]	33.67		39.42		33.60
Protein	32.91		38.67		32.74
Ligands/ions	47.47		59.56		48.47
Water	39.83		42.46		40.07
RMS deviations					
Bond lengths [Å]	0.002		0.003		0.004
Bond angles (°)	0.55		0.53		0.65
Ramachandran plot					
favored [%]	97.25		97.86		97.55
allowed [%]	2.45		2.14		2.45
outliers [%]	0.31		0.00		0.00

^1^ The numbers in brackets refer to the highest resolution shell.

## Supplementary Materials

The following are available online at http://www.mdpi.com/1424-8247/11/1/23/s1, Scheme S1: Synthesis of dibenzofurans 4 and **5**, Figure S1: Selectivity profiles of **4a** and **5**, Figure S2: IC_50_ values of **4a**, **4b** and **5** with CK2α and CK2α’ containing human CK2 holoenzyme, Figure S3: Morphological changes of ARPE19 and LNCaP cells induced by **4a**, **4b**, and **5**; PDB Codes.
