# Supplementary material for: A π-Halogen Bond of Dibenzofuranones with the Gatekeeper Phe113 in Human Protein Kinase CK2 Leads to Potent Tight Binding Inhibitors
Source: Pharmaceuticals (Basel). 2018 Feb 17;11(1):23. doi: 10.3390/ph11010023 (PMC5874719; doi:10.3390/ph11010023)
Supplement: Supplementary file 1 [file pharmaceuticals-11-00023-s001.pdf]

Supplementary Information

A π-Halogen Bond of Dibenzofuranones with the Gatekeeper Phe113 in Human Protein Kinase CK2 Leads to Potent Tight Binding Inhibitors

Alexander Schnitzler [1], Andreas Gratz [2], Andre Bollacke [2], Michael Weyrich [3], Uwe Kuckländer [4], Bernhard Wünsch [2], Claudia Götz [3], Karsten Niefind [1] and Joachim Jose [2,*]

[1] Department für Chemie, Institut für Biochemie, Universität zu Köln, Zülpicher Straße 47, D-50674 Köln, Germany; Alexander.Schnitzler@posteo.de (A.S.); karsten.niefind@uni-koeln.de (K.N.)
[2] Institut für Pharmazeutische und Medizinische Chemie, PharmaCampus, Westfälische Wilhelms-Universität Münster, Corrensstraße 48, D-48149 Münster, Germany; gratz.andreas@gmail.com (A.G.); andre.bo@web.de (A.B.); wuensch@uni-muenster.de (B.W.)
[3] Medizinische Biochemie und Molekularbiologie, Universität des Saarlandes, Kirrberger Str., Geb. 44, D-66421 Homburg, Germany; michaelweyrich@gmx.de (M.W.); claudia.goetz@uks.eu (C.G.)
[4] Institut für Pharmazeutische und Medizinische Chemie, Heinrich-Heine-Universität Düsseldorf, Universitätsstraße 1, D-40225 Düsseldorf, Germany; kucklaen@uni-duesseldorf.de
* Correspondence: joachim.jose@uni-muenster.de; Tel.: +49-251-8332-200

Listing of the contents of the files supplied as Supporting Information:

- Supplementary Scheme 1: Synthesis of dibenzofurans **4** and **5**
- Supplementary Figure 1: Selectivity profiles of **4a** and **5**
- Supplementary Figure 2: IC_{50} values of **4a**, **4b** and **5** with CK2α and CK2α' containing human CK2 holoenzyme
- Supplementary Figure 3: Morphological changes of ARPE19 and LNCaP cells induced by **4a**, **4b**, and **5**.
- PDB codes: **4a**: 5N9N; **4b**: 5N9L; **5**: 5N9K.

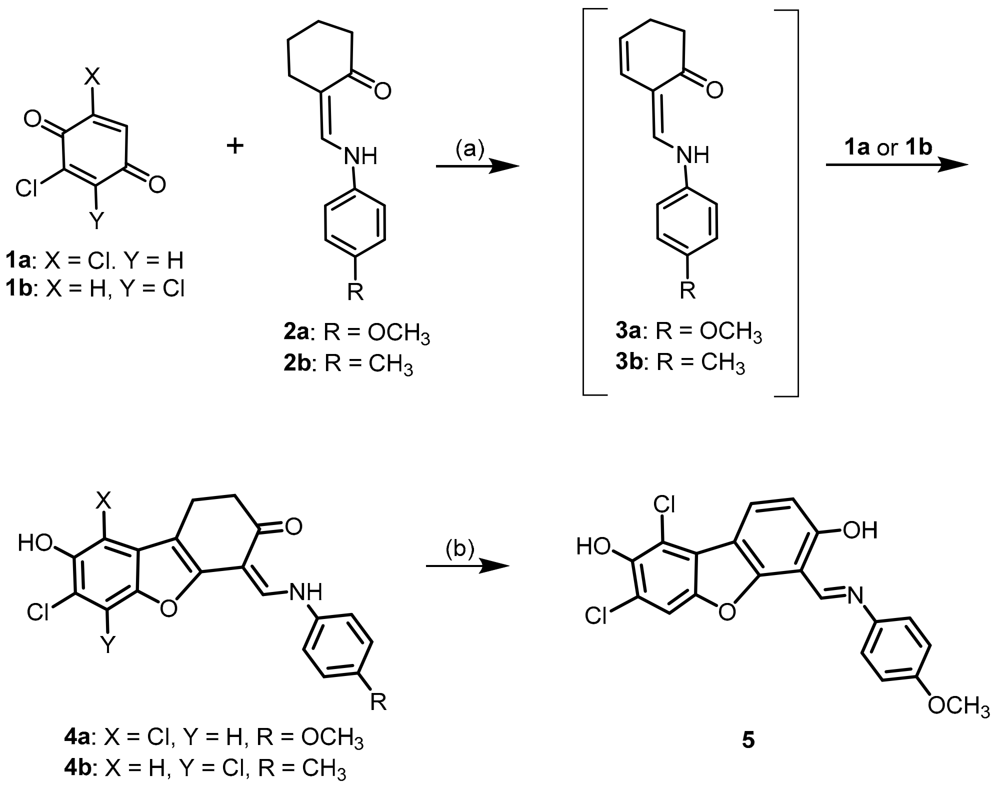

Supplementary Scheme 1: Synthesis of dibenzofurans **4 and 5.** Reagents and reaction conditions (a) and (b) are given in detail in the Experimental section.

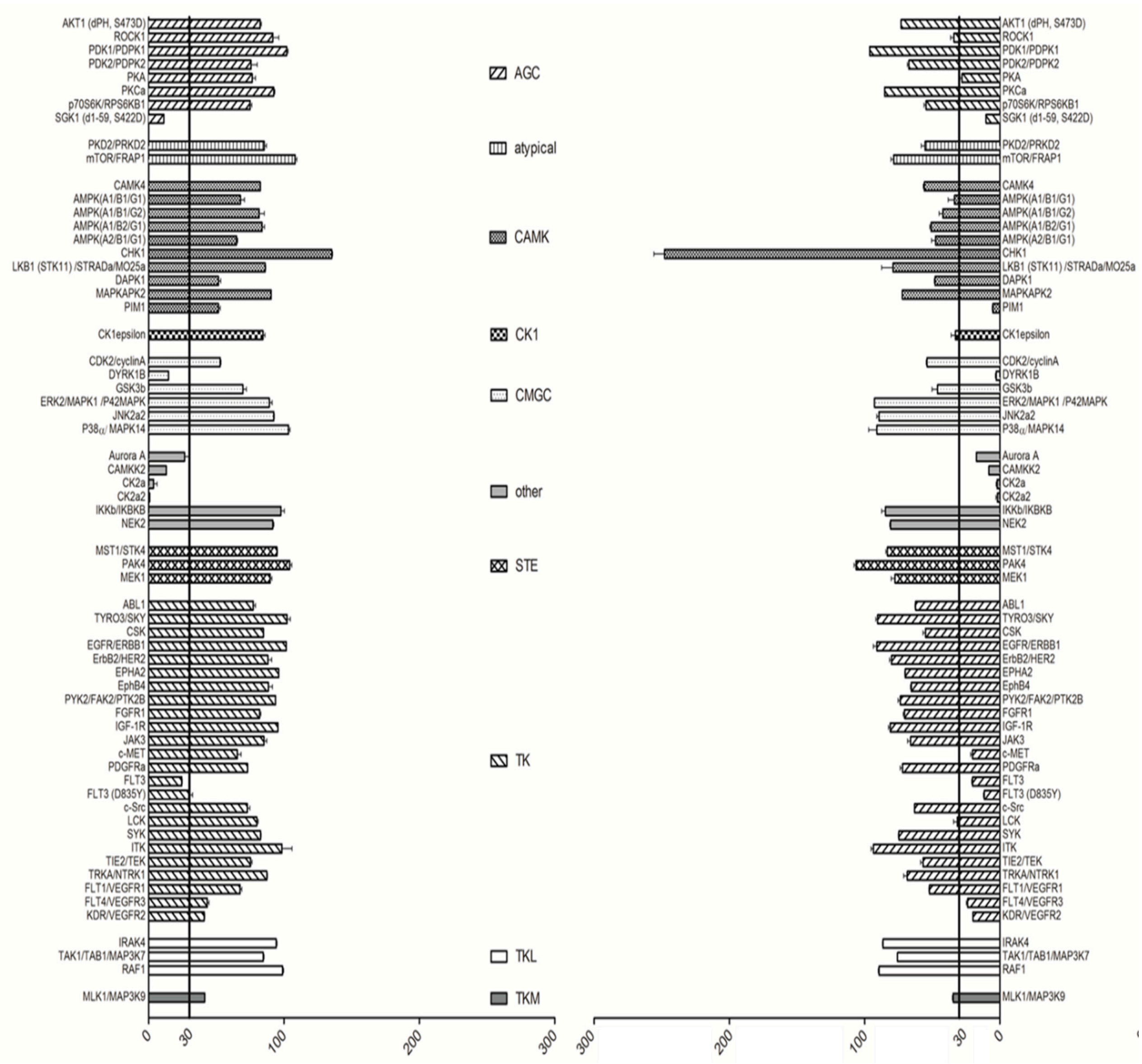

kinase activity in the presence of 10 μM compound 4a

kinase activity in the presence of 10 μM compound 5

Supplementary Figure S1: Selectivity profiles of **4a** and **5** against a panel of 61 human kinases from different families plus CK2α and CK2α'.

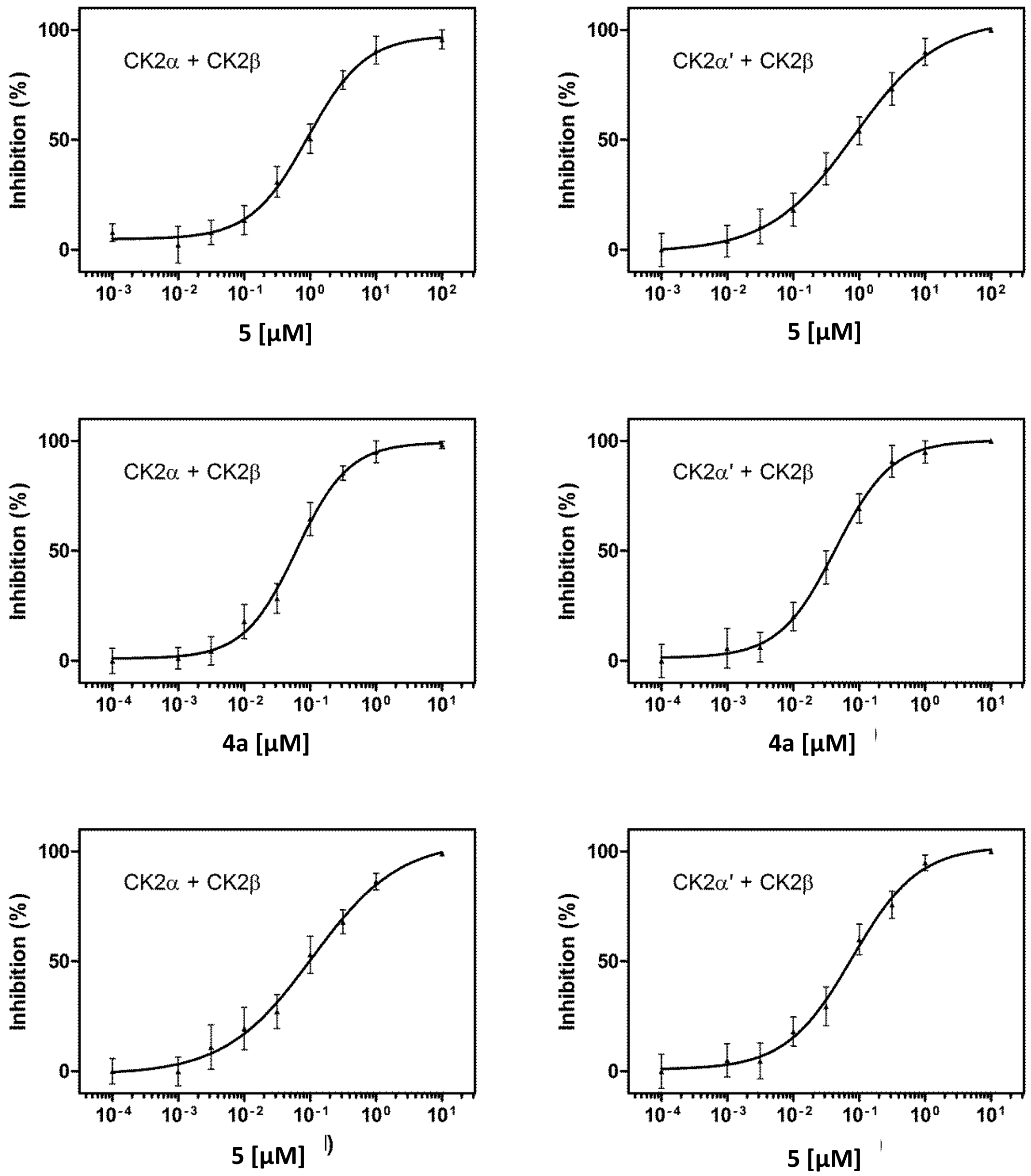

Supplementary Figure S2: IC_{50} determinations of **4a, 4b** and **5** with CK2α and CK2α' containing human CK2 holoenzyme displayed on *E. coli*. Dose-dependent inhibition of surface displayed CK2α + CK2β (left column) and CK2α' + CK2β (right column). IC_{50} were determined to be 0.877 µM and 0,746 µM for 4b (upper row), 0.062 µM and 0.043 µM for **4a** (middle row) and 0.098 µM and 0.073 µM for **5** (lower row). Mean values ± standard errors of the means (SEM) are shown.

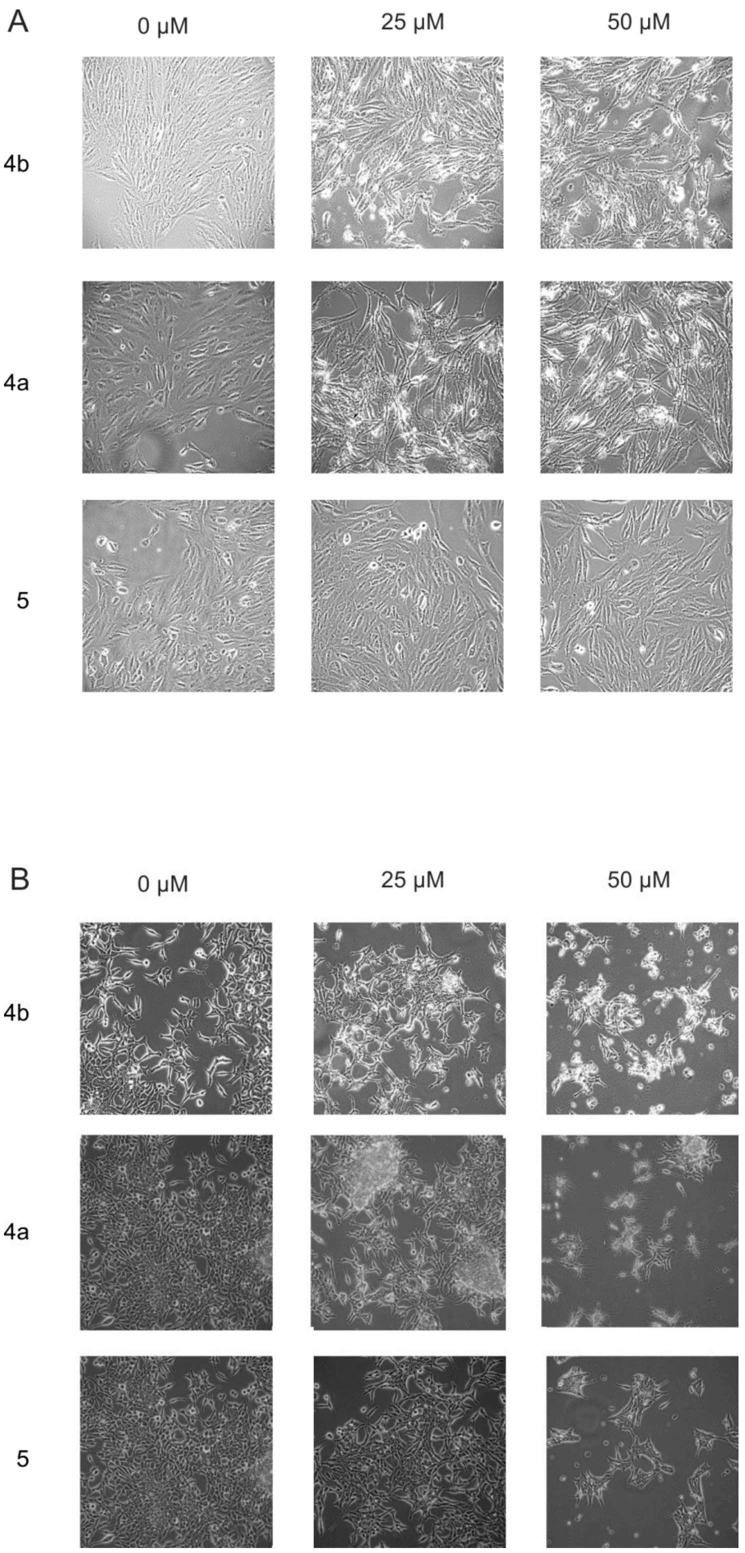

Supplementary Figure S3: Morphological changes of ARPE19 (A) and LNCaP (B) cells induced by dibenzofurans. ARPE19 and LNCaP cells were treated for 48 h with DMSO, 25 or 50 μM **4b**, **4a** or **5** and examined *via* phase contrast microscopy. Magnification: 400x.
